# Randomized trial examining the effect of exercise and wellness interventions on preventing postpartum depression and perceived stress

**DOI:** 10.1186/s12884-021-04257-8

**Published:** 2021-11-22

**Authors:** Beth A. Lewis, Katie Schuver, Shira Dunsiger, Lauren Samson, Amanda L. Frayeh, Carrie A. Terrell, Joseph T. Ciccolo, John Fischer, Melissa D. Avery

**Affiliations:** 1 University of Minnesota, School of Kinesiology, 1900 University Ave SE, Minneapolis, MN 55455 USA; 2 Center for Health Promotion and Health Equity in the Department of Behavioral and Social Sciences, Brown University, Box G-S121-4, Providence, RI 02912 USA; 3 Department of Obstetrics, Gynecology and Women’s Health, University of Minnesota, 604 24th Ave S, Ste 300, Minneapolis, MN 55454 USA; 4 Department of Biobehavioral Sciences, Teachers College Columbia University, 525 West 120th St., New York, NY USA; 5 University of Minnesota, School of Nursing, 308 Harvard St. SE, Minneapolis, MN 55455 USA

**Keywords:** Depression, Stress, Exercise, Postpartum depression

## Abstract

**Background:**

Approximately 13–19% of postpartum women experience postpartum depression and a majority report at least some stress during the postpartum phase. Traditional interventions such as psychotherapy and antidepressant medications are often not feasible or desirable. The purpose of this study was to examine two low cost, brief, accessible interventions designed to prevent postpartum depression and perceived stress among women at high risk.

**Methods:**

Participants (*n* = 450) who were on average four weeks postpartum, had a history of depression before pregnancy, and exercised less than 60 min per week were randomly assigned to one of the following three conditions: (1) 6-month telephone-based exercise intervention; (2) 6-month telephone-based wellness/support intervention (e.g., healthy eating, sleep, and perceived stress); or (3) usual care.

**Results:**

Overall, 2.4% of participants met criteria for depression at 6 months and 3.6% at 9 months with no differences between groups. At 6 months following randomization, median symptoms of depression were significantly lower among wellness participants compared to usual care participants (b = − 1.00, SE = 0.46, *p* = .03). Perceived stress at 6 months post-randomization was significantly lower among exercise vs. usual care participants (b = − 2.00, SE = .98, *p* = .04) and exercise vs. wellness participants (b = − 2.20, SE = 1.11, p = .04).

**Conclusions:**

The wellness intervention was efficacious for preventing symptoms of depression; however, postpartum depression that met the diagnostic criteria was surprisingly low in all conditions among this at risk sample of postpartum women. Exercise interventions may have a protective effect on perceived stress among women at risk for postpartum depression. Practitioners should consider integrating exercise and wellness interventions into postpartum care.

**Trial registration:**

Clinical Trials Number: NCT01883479 (06/21/2013).

## Background

Approximately 13–19% of women from middle and high-income countries experience postpartum depression following childbirth [1, 2]. The incidence of depression during pregnancy is similarly 12%, indicating that postpartum depression may start during pregnancy in some cases [3]. One study found that 3.2% of women have postpartum depression by one month postpartum suggesting that a majority of women develop postpartum depression following the first month [3]. The risk of developing postpartum depression is 20 times higher for women with a history of depression, compared to women without [4]. Prospective studies indicate that perceived stress, defined as the degree to which situations are appraised as stressful, is a significant risk factor for depression [5]. Thirty-seven percent of postpartum women report moderate to severe levels of stress [6]. Interpersonal and cognitive-behavioral psychotherapy is efficacious for treating and preventing postpartum depression [7, 8]; however, only 10% of women with postpartum depression seek treatment, likely due to the numerous barriers associated with psychotherapy including childcare, transportation, cost, stigma, and time constraints [9].

There is some evidence that antidepressants may be efficacious for severe cases of postpartum depression [10]; however, there is minimal support for the efficacy of antidepressant medication for mild to moderate levels of depression [10]. A recent Cochrane review identified only two low quality studies that examined the preventive effect of antidepressant medication on postpartum depression [11]. Additionally, postpartum women who breastfeed report reluctance to take antidepressant medication [12, 13]. Brief, low cost, highly accessible prevention interventions, such as exercise interventions, are needed to prevent both high levels of perceived stress and postpartum depression [9, 14, 15].

Research indicates that exercise is efficacious for treating depression among adults [16]. However, few high quality studies have examined the efficacy of exercise for postpartum depression [17]. A recent meta-analysis that identified 18 studies found that exercise was effective for preventing and reducing symptoms of depression among postpartum women. However, the effect sizes were small to moderate [18]. Subgroup analysis indicated higher treatment effects if the participant had a history of depression and/or elevated symptoms of depression. The studies had small samples sizes and were of low quality (e.g., majority lasted 12 weeks or less, lacked a diagnostic measure of depression, did not have a follow-up after the intervention ended). More research is needed to determine if the small effect sizes were due to the low quality of the studies or the lack of an effect of exercise on preventing postpartum depression.

One study that did examine the efficacy of a longer term home-based exercise intervention (i.e., 6 months) on postpartum depression found that women in the exercise intervention reported fewer symptoms of depression relative to the active wellness control condition [19]. One limitation is that women in the wellness control condition engaged in similar levels of exercise relative to the exercise condition. Therefore, the effect of exercise on postpartum depression was not adequately tested.

Since perinatal perceived stress is associated with an increased risk of symptoms of depression [20], it is also important to examine the effect of exercise and wellness interventions on stress levels. One study randomized postpartum women (*n* = 140) to a 12-week home-based exercise intervention (three times per week) or a routine care control. The exercise intervention was a progressive program focusing on both exercise and relaxation. Participants followed along to a video while exercising. Participants in the exercise condition reported lower levels of perceived stress when compared to the control [21]. Additional research with larger sample sizes and longer interventions are needed to examine the effect of exercise on perceived stress.

The purpose of this study was to examine the efficacy of exercise and wellness interventions on preventing postpartum depression and perceived stress. This study adds to the existing literature by including an adequately powered sample, diagnostic assessment of depression, and a long intervention including a follow-up three months after the intervention ended. Additionally, since exercise could have an impact on wellness related behaviors (e.g., eating, sleep habits), a wellness/support intervention that targeted health behaviors was included in order to isolate the effect of exercise alone on postpartum depression and perceived stress. Because previous studies indicated that active control groups may engage in exercise [19], a non-contact control arm was used in addition to the wellness control. We hypothesized that participants randomized to the exercise and wellness interventions would report lower rates of depression, symptoms of depression, and perceived stress than participants randomized to the usual care condition at 6 and 9 months following randomization. Additionally, we predicted that participants randomized to the exercise intervention would report lower rates of depression, symptoms of depression, and perceived stress than participants in the wellness/support condition at 6 and 9 months following randomization.

## Methods

### Study design

This was a randomized controlled single blind study conducted from January 2013 to August 2016 (MH096748). Participants (*n* = 450) were recruited from the community and randomized to one of the following three conditions each lasting 6 months: (1) Telephone-based exercise intervention (2) telephone-based wellness/support intervention; or (3) usual care. The effect of the interventions on meeting the criteria for depression, symptoms of depression (i.e., worry, lack of enjoyment in everyday activities, overwhelmed, worry, scared, self-blame, and sadness), and perceived stress was examined. Telephone-based interventions and assessments were chosen because they reduce the childcare, cost, time, and transportation issues related to face-to-face interventions but still include one-to-one counseling. Additionally, previous telephone-based exercise intervention trials with postpartum women have yielded high adherence [19, 22]. The intervention sessions and assessments were conducted over the telephone and consequently, there were no in-person sessions.

The randomization schedule was created by the study statistician using R (based on a permuted block randomization procedure with small, random-sized blocks). Randomizations were conducted by the project coordinator who was not involved in the assessments. This study was approved by the university’s institutional review board (IRB) and was performed in accordance with the ethical standard as stated by the 1964 Declaration of Helsinki and its later amendments. Participants completed the informed consent process including signing a written consent form. This trial is registered with clinicaltrials.gov (#NCT01883479; 06/21/2013).

### Participants

Postpartum women (average was 4.35 weeks post-birth at randomization; the latest a participant was randomized was 11 weeks postpartum) who had a history of depression were recruited from two large metropolitan areas in the upper Midwest. Exclusion criteria, which were assessed via a telephone-based interview, included the following: (1) No history of depression; (2) not willing to be randomized to any of the three study conditions; (3) less than 18 years of age (no upper limit for age); (4) currently exercising more than 60 min per week; (5) participating in another exercise or weight management study; (6) another person in the household participating in the study; (7) pre-existing hypertension or diabetes; (8) musculoskeletal problems that may interfere with exercising; (9) exercise induced asthma; (10) unable to exercise for 20 min continuously; (11) any condition that would make exercise unsafe or unwise; (12) taking medication that interferes with heart rate response to exercise such as beta blockers; (13) hospitalization for a psychiatric disorder in the past 6 months; and (14) currently receiving antidepressant medication or psychotherapy for depression. Because this was a prevention study, participants who were depressed at baseline were ineligible and referred to their healthcare provider. Participants who were depressed at baseline based on the Structured Clinical Interview for DSM-IV Axis I Disorders (SCID) were also excluded. History of depression was self-reported and defined as either being told by a healthcare provider that they have depression or having been prescribed medication for depression.

### Measures

The primary efficacy endpoint was the presence or absence of postpartum depression as determined by the Structured Clinical Interview for DSM-IV Axis I Disorders (SCID-I) [23]. The SCID-I is a semi-structured interview that is considered the gold standard for diagnosing postpartum depression. A master’s level research assistant with extensive training administered the SCID-I under the supervision of a licensed psychologist. Secondary endpoints included the 10-item Edinburgh Postnatal Depression Scale (EPDS), which assessed symptoms of depression [24]. The EPDS is rated on a 4-point Likert scale ranging from 0 to 3 (total score ranges from 0 to 30). Seven of the items are reverse scored and the scale is scored by adding the individual items together. The EPDS was specifically developed for postpartum women and has been shown to be valid [24]. Given even subclinical depression can be debilitating for new mothers [25] and in order to analyze varying levels of depressive symptoms, continuous scores on the EPDS were used for this study. The 14-item Perceived Stress Scale (PSS-14) [5], which assessed perceived stress, is rated on a five-point Likert scale ranging from 0 to 4 (total scores range from 0 to 56). Seven items are reverse scored and the total score is calculated by adding the items together. The PSS has been shown to be valid among the general population and the shorter version has been validated among postpartum women [5, 26].

To examine the success of the independent variable manipulation, exercise was assessed at 6 and 9 months following randomization using the 7-Day Physical Activity Interview (PAR) [27, 28] and ActiGraph (Fort Walton Beach, FL). The PAR is a semi-structured interview that assesses exercise over the previous seven days, which has been shown to be reliable and valid with postpartum women [29, 30]. Participants were instructed to wear the ActiGraph for seven days immediately prior to the 6 and 9 month post-randomization assessments. The ActiGraph is a small device that is worn at the waist that objectively measures exercise. Participants were mailed the ActiGraph and it was mailed back once it had been worn for seven days. Once it was received, the data was immediately downloaded for analysis. Studies indicate that the ActiGraph is reliable and valid [31–33]. For both the PAR and ActiGraph, exercise was considered any amount of activity that was at least of moderate intensity that lasted at least 10 min, which was the standard in the field at the time of the study. The activity was counted if it was in the moderate (55–70% of heart rate maximum; similar to a brisk walk) or vigorous intensity range (70–85% of heart rate maximum; similar to a jog) [34]. If there was less than 10 h of data or 60 continuous minutes of no activity, that data was excluded from the analysis [35]. Both the ActiGraph and PAR were used due to limitations related to the ActiGraph (e.g., low compliance to wearing the monitor, lost data as a result of malfunctioning, inaccuracy with certain types of exercise, and the monitor detaching from the clip) [36].

### Recruitment

Potential participants responded to advertisements by calling a study telephone line and eligibility was assessed over the telephone. The study was advertised via parenting magazines, targeted emails, online social media, and radio. Eligible and interested participants granted verbal consent over the telephone to contact their healthcare provider. They were also mailed a consent form and demographic questionnaire. Pregnant participants (72% of the sample) were instructed to call or email the study line once they gave birth. Healthcare provider consent, which was required by the Institutional Review Board to ensure that it was safe for the postpartum women to begin exercise, was obtained via fax after the participant gave birth. Specifically, the healthcare provider, who provided prenatal care for the participant, signed a consent form indicating that it was safe for the participant to start an exercise program. Participants next completed the baseline questionnaires and were administered the SCID. Because this was a prevention study, participants who met the criteria for postpartum depression at the randomization session were excluded and not randomized (*n* = 16). Eligible participants were randomized to one of the three study conditions at the end of this session. Participants completed a follow-up assessment at 6 and 9 months post-randomization by a research assistant blinded to study condition. At 9 months, participants in the usual care condition were given the option of receiving the exercise or wellness/support intervention.

### Interventions

The telephone-based exercise intervention and telephone-based wellness intervention consisted of 11 sessions (weekly during the first month, bi-weekly during months 2 and 3, and monthly for the next three months). The telephone sessions were delivered by trained health educators with master’s degrees who were supervised by a licensed psychologist. The goal of the exercise intervention was for participants to engage in exercise five days per week for at least 30 min per session. Exercise was defined as any activity lasting at least 10 min that was in the moderate or vigorous intensity range. Participants were taught how to take their heart rate and told to keep their heart rate in the moderate (55–70% of heart rate maximum which is estimated at 220 minus their age) or vigorous intensity range (70–85% of maximum heart rate) [34]. They were also told that the exercise sessions should be at least 10 min in length (could do three separate sessions in one day to obtain the 30 min recommendation) and feel at least like a brisk walk.

The exercise intervention was designed to motivate the participants to increase their exercise using strategies based on Social Cognitive Theory (SCT) [37] and the Transtheoretical Model (intervention content was tailored to how ready the participant was to increase exercise) [38]. Specific topics included exercise safety, benefits of exercise, social support, enjoyment of exercise, increasing self-efficacy, making exercise a habit, and exercise maintenance. The health educator followed a specific manual that addressed topics related to exercise; however, the intervention was tailored to the participant and any topic related to exercise could be discussed. The health educator processed emotions, thoughts, and behaviors related to exercise. Unlike general psychotherapy, a majority of the dialogue focused on exercise. This intervention has been shown to increase exercise to 130 min per week among postpartum women in previous trials [19, 39].

The wellness/support intervention addressed topics related to health and well-being including stress prevention, time management, healthy sleep, coping with fatigue, weight management, nutrition, and healthy home topics (i.e., creating a safe home for baby that could help reduce stress for the participant). To control for non-specific factors such as rapport and empathy, the same health educators were used for the exercise and wellness/support interventions. The health educators provided support to the participants through active listening and empathy. The health educators engaged in problem solving strategies to help reduce the impact of stressors that typically occur during the postpartum phase. The health educators did not discuss exercise with participants in the wellness/support condition. Participants in the wellness/support intervention received mailings addressing health and wellness/support topics on the same schedule as participants in the exercise intervention.

One-third of the participants had at least one exercise or wellness session recorded and 10% of the assessment sessions were recorded. These recordings were reviewed by a doctoral-level health educator to ensure that the health educators adhered to the intervention protocol and to assess for non-specific counseling strategies. The health educators were trained to respond to the needs of the participants. Therefore, empathy, activity listening, and rapport were rated on a five-point Likert scale for each session. More information regarding the study protocol can be found elsewhere [40].

### Data analysis

Baseline demographic variables were summarized for the aggregate sample and compared between-conditions using analysis of variance (ANOVA) for continuous variables and Chi-Square tests for categorical variables. The distribution of each of the outcome variables was explored using graphical methods, and in the case of non-normal outcomes, transformations were used to attempt to bring data towards normality. Correlations between self-reported and objectively measured exercise were assessed over time using Spearman Rank Correlations (which are less subject to outlying values).

Effects of the intervention on postpartum depression status as measured by the SCID-I was assessed using a longitudinal regression model implemented with Generalized Estimating Equations (GEE’s) with robust standard errors. The models included effects of treatment and time, as well as the interaction between them. Standard errors were adjusted for repeated measures of the outcome over time within participant. For symptoms of depression, perceived stress, and self-reported exercise, effects of the treatment group were assessed using a series of longitudinal quantile regression models, which model the median outcome (as opposed to the mean) over time. Quantile models are more appropriate for variables that fail to meet normality assumptions (even after transformation).

Primary outcomes analyses were run including all randomized participants, following an intent-to-treat model. Estimation was carried out using a likelihood-based approach and thus made use of all available data without directly imputing missing outcomes. As we recognize this approach assumes the data is missing at random (MAR), we compared the findings to a multiple imputation approach. With no significant differences in effects for primary, secondary or predictor/moderator models, we present the results under a MAR assumption.

All analyses were run in SAS 9.3 and STATA SE 15, with alpha set a priori at .05. The sample size calculation was based on the assumption of 80% power for both postpartum depression and symptoms of depression. Estimates were based on a previous trial [19]. The power analysis estimated 150 participants needed for each arm at 6 months assuming a 10% attrition rate.

## Results

### Participant flow

**Fig. 1:**
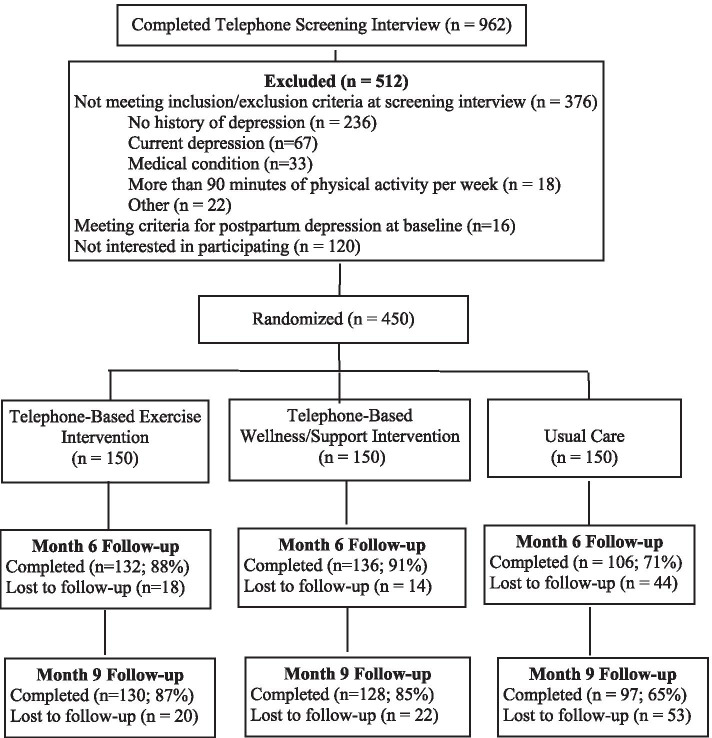
Trial Flowchart

There were 450 participants randomized to the three conditions (150 to each condition; see Fig. 1). The average age was 31 years and a majority were Caucasian, married, and had a college degree (see Table 1 for demographic data). It was the first child for approximately one-third of the sample. There were significant between-group differences for baseline age (*p* = .01) and symptoms of depression (*p* = .03). Specifically, participants in the exercise condition were younger than participants in the wellness condition and older than participants in the usual care condition. Participants in the exercise condition also reported fewer symptoms of depression at baseline than participants the wellness condition. These variables were included as covariates in subsequent models. Ten percent reported starting medication for depression (13% of exercise participants, 11% of wellness participants, and 7% of usual care), 14% received psychotherapy after enrolling in the study (15% of exercise participants, 14% of wellness participants, and 14% of usual care), and 5% received both medication for depression and psychotherapy (5% of exercise participants, 5% of wellness participants, and 5% of usual care). There were no differences between group for starting medication for depression or receiving psychotherapy.

**Table 1:** Baseline Characteristics by Study Arm

Demographic Variable	Total (*N* = 450)	Exercise (*n* = 150)	Wellness (*n* = 150)	Usual Care (n = 150)
Age, mean (SD), y	30.74 (5.14)	31.03 (4.68)	31.44 (5.29)	29.77 (5.32)
Race, n (%)				
Caucasian	327 (72.7)	118 (78.7)	103 (68.7)	106 (70.7)
African-American	53 (11.8)	10 (6.7)	21 (14.0)	22 (14.7)
Other	78 (15.5)	22 (14.7)	26 (17.3)	22 (14.7)
Marital Status, n (%)				
Married	322 (75.2)	113 (78.5)	116 (79.5)	93 (67.4)
Single	90 (21.0)	26 (18.1)	26 (17.8)	38 (27.5)
Divorced	13 (3.0)	4 (2.8)	4 (2.7)	5 (3.6)
Separated	3 (0.7)	1 (0.7)	0	2 (1.4)
Education Level, n (%)				
Less than high school graduate	7.1 (1.6)	0 (0)	2 (1.4)	5 (3.6)
High school graduate	25 (5.8)	5 (3.5)	10 (6.8)	10 (7.2)
Some college	93 (21.7)	31 (21.5)	27 (18.5)	35 (25.4)
College graduate	181 (42.3)	64 (44.4)	64 (44.5)	52 (37.7)
Post-graduate work	122 (28.5)	44 (30.6)	42 (22.8)	36 (26.1)
Household Income, n (%)				
Under 10,000	27 (6.3)	3 (2.1)	6 (4.1)	18 (13.0)
Between $10,000 and 19,999	24 (5.6)	3 (2.1)	12 (8.2)	9 (6.5)
Between $20,000 and 29,999	36 (8.4)	17 (11.8)	9 (6.2)	10 (7.2)
Between $30,000 and 39,999	42 (9.8)	16 (11.1)	15 (10.3)	11 (8.0)
Between $40,000 and 50,000	46 (10.7)	14 (9.7)	12 (8.2)	20 (14.5)
Over $50,000	238 (55.6)	85 (59.0)	89 (61.0)	64 (46.4)
Don’t know/refuse	15 (3.5)	6 (4.2)	3(2.1)	4.77 (2.35)
First Biological Child, n (%)	136 (32.0)	47 (33.1)	41 (28.3)	48 (34.8)

### Exercise adherence

Women in the exercise condition reported more exercise minutes per week than women in the usual care condition at 6 months after randomization (b = 40.00, SE = 14.66, *p* = .01; see Table 2). There was a trend at 9 months post-randomization for women in the exercise condition to report more exercise than women in usual care (*p* = .09). The ActiGraph (objective measure of exercise) significantly correlated with the PAR at 6 months post-randomization, rho = .31, *p* < .001, and there was a trend at 9 months post-randomization, rho = .17, *p* = .07.

**Table 2:** Average Exercise Minutes per Week by Study Arm at 6 and 9 Months

	Exercise (n = 150) mean (SD)	Wellness (n = 150) mean (SD)	Usual Care (n = 150) mean (SD)	*P* Value
**MVPA**^a^				
6 months	125.40 (91.16)	122.14 (104.60)	107.71 (81.63)	0.10
9 months	133.02 (105.09)	121.29 (90.18)	115.26 (115.64)	0.33

*Abbreviation*: *MVPA* Moderate to Vigorous Intensity activity based on the 7-Day Physical Activity Recall Interview; SD, Standard Deviation

^a^Reported as minutes per week

### Primary outcomes

Unadjusted summaries of the outcomes variables over time by group are presented in Table 3. There were no significant differences across the three groups for meeting the diagnostic criteria for postpartum depression at either timepoint (based on the SCID). Adjusted models suggest that at 6 months, median depressive symptoms were significantly lower among wellness participants compared to usual care (b = − 1.00, SE = 0.46, *p* = .03). There were no differences between wellness and usual care at 9 months. There were also no differences between exercise and usual care and exercise and wellness on depressive symptoms at 6 or 9 months. Median perceived stress at 6 months was significantly lower among exercise vs. usual care participants (b = − 2.00, SE = .98, *p* = .04) and exercise vs. wellness (b = − 2.20, SE = 1.11, p = .04). There were no differences between wellness and usual care at 6 months. There were no differences on median perceived stress between any of the group at 9 months.

**Table 3:** Unadjusted Dependent Variables of Depression Symptoms and Perceived Stress Over Time by Group

Dependent Variable	Total Sample (N = 450)	Exercise (n = 150)	Wellness (n = 150)	Usual Care (n = 150)	*P Value*
**Depressed (SCID), n (%)**					
6 months	11 (2.4)	4 (2.7)	2 (1.3)	5 (3.3)	.52
9 months	16 (3.6)	4 (2.7)	8 (5.3)	4 (2.7)	.36
**Edinburgh Postnatal Depression Scale, median (range)**^**a**^					
Baseline	8 (19)	7 (14)	9 (19)	8 (19)	.14
6 months	7 (21)	7 (19)	7 (18)	7 (20)	.69
9 months	7 (19)	7 (17)	7 (19)	7 (17)	.25
**Perceived Stress Scale, median (range)**^**b**^					
Baseline	24 (41)	24 (33)	25 (38)	25 (39)	.20
6 months	22 (39)	20 (38)	23 (30)	22 (36)	.07
9 months	22 (40)	22 (36)	23 (37)	22 (39)	.25

*Abbreviations*: *SCID* Structured Clinical Interview for DSM-IV Axis I Disorders

^a^The possible range of scores was 0–30 for the EPDS

^b^The possible range of scores was 0–56 for the PSS

## Discussion

There were no differences among the number of women in the three conditions who met the diagnostic criteria for postpartum depression at 6 or 9 months after initiation of the study. The rate of postpartum depression was surprisingly lower than expected for postpartum women with a history of depression [4]. Specifically, 2.4% of participants met the diagnostic criteria for postpartum depression at 6 months; however, it would be expected that approximately one-third of participants would meet the diagnostic criteria for postpartum depression based on previous studies among women who have a history of depression [9]. This finding is contrary to a recent meta-analysis indicating that light to moderate intensity physical activity leads to improved symptoms of depression [18].

Perhaps individuals choosing to participate in the study were more aware of their high risk for postpartum depression than those not participating and engaged in preventive efforts in addition to the study, which decreased their risk for postpartum depression. Symptoms of depression include social isolation, apathy, lack of initiative, lack of concentration, and loss of social connection. If present, these symptoms could impede women’s response to a study advertisement in the community. Women with depression, subclinical symptoms of depression, or even a propensity for developing depression may be less likely to take initiative to participate in a research study that requires a significant amount of energy and/or concentration. Therefore, the sampling method used in this study, which required a high level of initiative, could have resulted in the overall sample reporting fewer symptoms of depression.

Even though participants in the exercise intervention engaged in higher levels of exercise than participants in usual care, similar to previous studies [19], women in the wellness/support intervention did engage in 121 min of exercise per week and participants in usual care participated in 98 min per week at 6 months. Exercise levels were maintained in all three conditions at 9 months and even slightly increased in the exercise and usual care conditions, although the increases were not significant. Therefore, it is possible that exercise played a role in preventing postpartum depression among women in the wellness/support and usual care conditions, as evidenced by the low rate of depression based on the SCID across all three conditions at both six and nine months.

Symptoms of depression as measured by the Edinburgh Postnatal Depression Scale (EPDS) at 6 months were significantly lower among participants randomized to the wellness intervention relative to those in the usual care condition at 6 but not 9 months following randomization. The 6 month EPDS finding is inconsistent with the SCID finding. This discrepancy perhaps occurred because the EPDS produces a continuous score and the SCID is a diagnostic interview for postpartum depression (yes/no). Therefore, unlike the SCID, the EPDS provides information on symptoms of depression. The wellness intervention focused on healthy sleep and perceived stress reduction, both of which could decrease the risk for symptoms of depression [41, 42]. However, inconsistent with our hypotheses and previous studies [18], symptoms of depression were not lower among participants in the exercise intervention relative to participants in usual care at either timepoint.

Even though the median scores for the EPDS were below the clinical threshold for depression (median scores ranged from 7 to 9 across the groups), there is evidence that even subthreshold scores are related to postpartum difficulties. For example, research indicates that scores in the 6 to 8 range are related to sleep disturbances, social isolation, and maternal stress [25]. Therefore, even mothers who were not classified as depressed based on the EPDS, could still have significant problems during the postpartum period.

As hypothesized, perceived stress was significantly lower among women in the exercise intervention relative to women in either the wellness or usual care conditions at 6 months following randomization. This is consistent with a previous study examining the effect of a four-week exercise intervention on perceived stress [21]. It is interesting that participants in the exercise condition reported lower stress levels but not lower depressive symptoms relative to the usual care condition. It is possible that exercise alone is enough to impact stress but a wellness intervention in addition to exercise is needed to impact depression. Perhaps this is due to that fact that stress can be a precursor to depression [41] and the added complexity of depression relative to stress. It is possible that the combination of exercise and wellness would significantly impact depression. It is important to note that there were no differences between the exercise and wellness conditions on minutes of exercise per week (both groups exercised approximately 120–130 min per week) further supporting the idea that wellness in combination with exercise may be particularly important for decreasing depression.

Regarding at 9 months following randomization, there were no differences among any of the study arms on depression, symptoms of depression, or perceived stress. The symptoms of depression and perceived stress scores for all groups returned to baseline levels for both measures at 9 months, which likely accounted for the lack of differences. It is possible that as the newborn ages, variables such as perceived stress levels and sleep quality return to normal, which accounts for the lack of differences at 9 months.

Strengths of this study included the use of a diagnostic interview for depression, large sample size, and a relatively diverse sample. Additionally, effect sizes were in the moderate range indicating potential clinical significance. Despite these strengths, there are several limitations. First, despite randomization, symptoms of depression at baseline was significantly higher among women in the wellness/support condition when compared to the other two groups. Second, history of trauma was not assessed in this study, which is another limitation since previous trauma can increase the risk for postpartum depression [43].

Third, the low rate of depression at 6 and 9 months post-randomization may have been due to excluding participants who met the criteria for postpartum depression at the screening interview and baseline. Research indicates that approximately 3.4% of women are depressed by one month postpartum [3], which is consistent with the number of participants excluded from our study at baseline (3.6% were excluded at baseline due to depression). Excluding high risk participants at baseline may have contributed to the low rates of depression. Additionally, the mean age was relatively high, which may have contributed to being well prepared for the postpartum phase; thus, reducing the risk for postpartum depression.

Ten percent of the participants started an antidepressant and 14% participated in psychotherapy, which also may have impacted the overall rates of depression and stress. However, there was no significant differences between groups on psychotherapy or antidepressant use and consequently, it is unlikely that psychotherapy and/or antidepressant use influenced the differences between groups on the outcomes. Participants were randomized on average at 4 weeks postpartum; however, the range was up to 11 weeks, which may have also impacted the results.

A fourth limitation is that a majority of potential participants responded to an advertisement. Therefore, it is likely that the sample consisted of motivated individuals who were not representative of the general population. A fifth limitation is that the primary measure for exercise was self-report due to the low compliance rate with wearing the ActiGraph monitor and issues with the ActiGraph detaching from the clip (valid ActiGraph data was obtained for 54% of the sample at 6 months and 29% at 9 months with no differences between groups). It is possible that participants either underreported or over-reported their exercise. Finally, it is unclear if this study would generalize to participants without a history of depression.

Future studies that examine the efficacy of exercise interventions during pregnancy are needed. This would allow women to establish exercise as a habit during pregnancy, which could assist with the transition to exercising in the postpartum phase. Since history of depression is a significant risk factor for prenatal depression [44], prevention efforts may be particularly beneficial if started during pregnancy. Future studies should also explore the effect of various types of exercise on postpartum depression. Perhaps the social component of exercising in a group would appeal to this population of women. Therefore, future studies could compare the efficacy of exercise interventions that integrate social support to those interventions that do not. Additionally, high intensity interval training, which has the benefit of being time efficient [45], may be especially beneficial for this population once medically cleared to engage in high intensity exercise. It is possible that exercise alone is not enough to impact depression but if combined with a wellness intervention, it can impact depression. Finally, this study was conducted with participants who had a history of depression in order to target an at-risk population. It is possible that exercise and wellness interventions would impact postpartum women without a history of depression differently and therefore, studies among these individuals should be conducted in the future.

## Conclusions

In summary, the purpose of this study was to examine the efficacy of exercise and wellness interventions for preventing postpartum depression and perceived stress. Results indicated that depression was surprisingly low among women in all three conditions among this at risk sample of postpartum women. No support for the preventive effect of exercise on postpartum depression was found; however, exercise may help lower perceived stress levels among women at risk for postpartum depression. Additionally, wellness interventions may be efficacious for lowering symptoms of depression. Because this study was large and adequately powered to detect differences between groups, it is possible that exercise alone is not enough to prevent postpartum depression. Because depression can start during pregnancy, more research is needed that targets depression during both pregnancy and postpartum. Studies that start an intervention during pregnancy may prevent depression from occurring during pregnancy, which could lead to a reduced risk of postpartum depression. Although there was no effect of exercise on preventing postpartum depression, practitioners should still integrate exercise into postpartum care given the general health benefits associated with exercise and its potential impact on perceived stress.

## Data Availability

The datasets used and/or analysed during the current study are available from the corresponding author on reasonable request.
